# Association of Dietary Acid Load with the Prevalence of Metabolic Syndrome among Participants in Baseline Survey of the Japan Multi-Institutional Collaborative Cohort Study

**DOI:** 10.3390/nu12061605

**Published:** 2020-05-29

**Authors:** Kokichi Arisawa, Sakurako Katsuura-Kamano, Hirokazu Uemura, Nguyen Van Tien, Asahi Hishida, Takashi Tamura, Yoko Kubo, Mineko Tsukamoto, Keitaro Tanaka, Megumi Hara, Toshiro Takezaki, Daisaku Nishimoto, Teruhide Koyama, Etsuko Ozaki, Sadao Suzuki, Takeshi Nishiyama, Kiyonori Kuriki, Aya Kadota, Naoyuki Takashima, Hiroaki Ikezaki, Masayuki Murata, Isao Oze, Keitaro Matsuo, Haruo Mikami, Yohko Nakamura, Kenji Takeuchi, Kenji Wakai

**Affiliations:** 1 Department of Preventive Medicine, Tokushima University Graduate School of Biomedical Sciences, Tokushima 770-8503, Japan; skamano@tokushima-u.ac.jp (S.K.-K.); uemura.hirokazu@tokushima-u.ac.jp (H.U.); tiennv@tbump.edu.vn (N.V.T.); 2 Department of Preventive Medicine, Nagoya University Graduate School of Medicine, Nagoya 466-8550, Japan; a-hishi@med.nagoya-u.ac.jp (A.H.); ttamura@med.nagoya-u.ac.jp (T.T.); protonk@med.nagoya-u.ac.jp (Y.K.); tsukamoto.mineko@b.mbox.nagoya-u.ac.jp (M.T.); k.takeuchi@med.nagoya-u.ac.jp (K.T.); wakai@med.nagoya-u.ac.jp (K.W.); 3 Department of Preventive Medicine, Faculty of Medicine, Saga University, Saga 849-8501, Japan; tanakake@cc.saga-u.ac.jp (K.T.); harameg@cc.saga-u.ac.jp (M.H.); 4 Department of International Island and Community Medicine, Kagoshima University Graduate School of Medical and Dental Sciences, Kagoshima 890-8544, Japan; takezaki@m.kufm.kagoshima-u.ac.jp; 5 School of Health Sciences, Faculty of Medicine, Kagoshima University, Kagoshima 890-8544, Japan; daisaku@health.nop.kagoshima-u.ac.jp; 6 Department of Epidemiology for Community Health and Medicine, Kyoto Prefectural University of Medicine, Kyoto 602-8566, Japan; tkoyama@koto.kpu-m.ac.jp (T.K.); ozaki@koto.kpu-m.ac.jp (E.O.); 7 Department of Public Health, Nagoya City University Graduate School of Medical Sciences, Nagoya 467-8601, Japan; ssuzuki@med.nagoya-cu.ac.jp (S.S.); psychogenomics@gmail.com (T.N.); 8 Laboratory of Public Health, Division of Nutritional Sciences, School of Food and Nutritional Sciences, University of Shizuoka, Shizuoka 422-8526, Japan; kuriki@u-shizuoka-ken.ac.jp; 9 Department of Public Health, Shiga University of Medical Science, Otsu, Shiga 520-2192, Japan; ayakd@belle.shiga-med.ac.jp (A.K.); n.takashima@med.kindai.ac.jp (N.T.); 10 Department of Public Health, Faculty of Medicine, Kindai University, Osaka-Sayama, Osaka 589-8511, Japan; 11 Department of Comprehensive General Internal Medicine, Faculty of Medical Sciences, Kyushu University Graduate School of Medicine, Fukuoka 812-8582, Japan; ikezaki@gim.med.kyushu-u.ac.jp; 12 Department of General Internal Medicine, Kyushu University Hospital, Fukuoka 812-8582, Japan; mmurata@gim.med.kyushu-u.ac.jp; 13 Division of Cancer Epidemiology and Prevention, Aichi Cancer Center Research Institute, Nagoya 464-8681, Japan; i_oze@aichi-cc.jp (I.O.); kmatsuo@aichi-cc.jp (K.M.); 14 Department of Cancer Epidemiology, Nagoya University Graduate School of Medicine, Nagoya 466-8550, Japan; 15 Cancer Prevention Center, Chiba Cancer Center Research Institute, Chiba 260-8717, Japan; hmikami@chiba-cc.jp (H.M.); ynakamur@chiba-cc.jp (Y.N.)

**Keywords:** dietary acid load, metabolic syndrome, net endogenous acid production, cross-sectional study

## Abstract

The association between dietary acid load and metabolic syndrome (MetS) has not been fully investigated. A cross-sectional study was performed on 14,042 men and 14,105 women (aged 35–69 years) who participated in a baseline survey of the Japan Multi-Institutional Collaborative Cohort study. Dietary acid load was assessed using the net-endogenous-acid-production (NEAP) score that is closely correlated with the rate of renal net acid excretion. MetS was diagnosed according to the Joint Interim Statement Criteria of 2009 using body-mass index instead of waist circumference. After adjusting for potential confounders, higher NEAP scores were associated with a significantly increased odds ratio (OR) of MetS, obesity, high blood pressure, and high fasting blood glucose. These associations remained significant after further adjustment for carbohydrate intake or two nutrient-pattern scores significantly associated with MetS. After adjustment for fiber, iron, potassium, and vitamin pattern scores, the OR of MetS for the highest quartile of NEAP scores, relative to the lowest quartile, was 1.25 (95% confidence interval 1.12–1.39). There was no significant interaction between sex, age, or body-mass index and NEAP. Higher dietary acid load was associated with a higher prevalence of MetS and several of its components, independently of carbohydrate intake or nutrient patterns.

## 1. Introduction

Metabolic syndrome (MetS) is characterized by a cluster of high blood pressure, central obesity, high serum triglyceride levels, low serum high-density lipoprotein (HDL) cholesterol levels, and high fasting blood glucose, with insulin resistance as an underlying condition [1]. People who have MetS are at increased risk of the future development of Type 2 diabetes and cardiovascular diseases [2,3].

In addition to excessive energy intake and a sedentary lifestyle, dietary quality is closely linked to MetS. A prudent/healthy diet pattern, characterized by high intake of vegetables, fruits, whole grains, and legumes, was inversely associated with MetS, while a Western/unhealthy pattern, characterized by a high intake of meat, processed meat, refined grains, and sweets, was positively associated with MetS in meta-analysis of 28 cross-sectional studies [4]. The dietary-approaches-to-stop-hypertension (DASH) [5] and Mediterranean diets [6] were associated with reduced risk of MetS.

Recently, positive associations of low-grade metabolic acidosis, assessed by a high dietary acid load [7,8,9] or high animal-protein intake [10], with increased risk of Type 2 diabetes, were reported from Europe, Japan, and the United States (U.S.). In addition, low serum bicarbonate levels, a high anion gap, and a high dietary acid load were reported to be associated with insulin resistance [11,12]. Dietary acid load was also associated with high blood pressure [13,14,15], obesity and high serum low-density lipoprotein (LDL) cholesterol levels [16].

As indices of dietary acid load, potential renal acid load (PRAL) [17] and net endogenous acid production (NEAP) [18] have been frequently used in epidemiological studies. Generally, PRAL and NEAP are positively correlated with intake of meat, fish, and eggs, and inversely correlated with an intake of vegetables, fruits, and dairy products [7,8,16]. Therefore, it is thought that PRAL and NEAP are positively correlated with a Western/unhealthy diet pattern, and negatively correlated with a prudent/healthy diet pattern. This suggests that dietary patterns become potential confounders when assessing the relationship between dietary acid load and MetS. Another concern is whether observed associations of dietary quality with MetS are mediated by dietary acid load.

Only a small number of studies have examined the association between dietary acid load and MetS, except for small studies on patients with Type 2 diabetes [19] or Iranian women [20]. In these two earlier studies, lifestyle factors, including dietary habits, were not taken into consideration. In the present study, we examined the associations of NEAP with MetS and its components among approximately 28,000 participants in a baseline survey of the Japan Multi-Institutional Collaborative Cohort (J-MICC) study, considering lifestyle factors including nutrient patterns.

## 2. Materials and Methods

### 2.1. Study Subjects

The population for the present study consisted of Japanese men and women aged 35 to 69 years who took part in the baseline survey of the J-MICC study. The purpose and outline of the J-MICC study were described in a previous report [21]. In short, the aim of the J-MICC study was to prospectively investigate the interactions of lifestyle and genetic factors with the risk of chronic diseases. Written informed consent was obtained from each participant after the outline and purposes of this study were explained. This study was performed in accordance with the Declaration of Helsinki, and the study protocol was approved by the institutional review boards of Nagoya University Graduate School of Medicine (IRB no. 2010-0939-7), Aichi Cancer Center Research Institute (IRB no. 2016-2-10), Tokushima University Hospital (IRB no. 466-2), and all other participating study centers.

**Figure 1:**
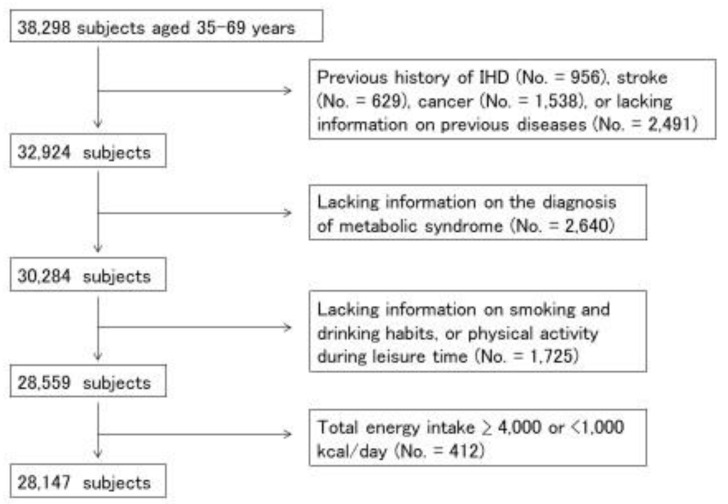
Selection-process flowchart for study subjects. Among 38,298 participants in baseline survey of the Japan Multi-Institutional Collaborative Cohort (J-MICC) study, 28,147 subjects (14,042 men and 14,105 women) were analyzed.

Participants from seven study centers that used the same questionnaires and performed examination of blood glucose were included (no. = 38,298, dataset of version 2019.7.29). We excluded participants who had a history of ischemic heart disease (IHD), stroke, or cancer, or who lacked information on previous diseases (No. = 5374), information needed for the diagnosis of MetS (no. = 2640), smoking and drinking habits, physical activity during leisure time (no. = 1725), or whose total energy intake was extremely high or low (≥4000 or <1000 kcal/day) (no. = 412) (Figure 1). A total of 28,147 participants (14,042 men and 14,105 women) were used for statistical analysis.

### 2.2. Food-Frequency Questionnaire (FFQ) and Covariates

Study subjects were requested to fill out a self-administered questionnaire about dietary habits, history of current and previous diseases, medication and supplement consumption, physical activity during leisure time, ways of coping with stress, and smoking and drinking habits. A validated short FFQ was used to assess dietary habits [22,23]. Participants were asked how often they had consumed 46 foods and beverages over the previous year. Frequency of consumption of staple foods, i.e., rice, bread, and noodles, at each of breakfast, lunch, and dinner were divided into six categories from rarely to every day. Information on portion size (how many cups, slices, or pieces) was also collected. For the 43 other foods and beverages, intake frequency was categorized into eight categories from rarely to ≥3 times per day. Intake of total energy and 26 nutrients were estimated using a program developed by the Department of Public Health, Nagoya City University School of Medicine. A validation study was performed by comparing the intake of total energy and 26 nutrients, estimated on the basis of the FFQ and 3-day-weighted diet records. Deattenuated, log-transformed, and energy-adjusted correlation coefficients for the intake of total energy and the 26 nutrients ranged from 0.10 to 0.86 [22]. For potassium, the correlation coefficient between 12-day-weighted diet records and FFQ was 0.52 for men and 0.45 for women. This FFQ also showed good one-year interval reproducibility for the consumption of foods and nutrients [23].

Physical activity during leisure time was calculated by multiplying frequency (five categories from never to ≥5 times/week) and average duration (six categories from ≤30 min to ≥4 h) of light (such as walking, hiking, and golfing at 3.4 metabolic equivalents (METs)), moderate (such as jogging, swimming, skiing, and dancing at 7.0 METs), and vigorous-intensity exercises (such as marathon running, ball games, and combat sports at 10.0 METs). The three levels of exercise were added and expressed as MET hours/week. Body-mass index (BMI) was calculated as weight (kg) divided by the square of height (m^2^). Smoking status was classified into three groups, namely, current smoker, past smoker, and never. Drinking status was categorized as current drinker, past drinker, and never. Academic career was classified as elementary school/junior high school, high school, professional school, junior college/technical college, university/college, graduate school, and other.

### 2.3. Metabolic-Syndrome Diagnosis

MetS was diagnosed according to the Joint Interim Statement Criteria of 2009, with a modification [1]. Since waist circumference was not measured in some study sites, BMI was used. BMI is closely correlated with abdominal circumference, and the most accurate BMI cut-off point for large abdominal circumference (90 cm for men and 80 cm for women, the cut-off point for the diagnosis of MetS for Asians) was approximately 25 kg/m^2^ [24]. Subjects who had at least three of the five following conditions were judged as having MetS: BMI ≥ 25 kg/m^2^, blood pressure ≥ 130/85 mmHg or treatment for hypertension, serum HDL cholesterol level < 40 mg/dL for men and <50 mg/dL for women, serum triglycerides ≥ 150 mg/dL, and fasting blood glucose level ≥ 100 mg/dL or treatment for diabetes.

### 2.4. Dietary-Acid-Load Estimation

NEAP was estimated using the following formula [18]:

NEAP (mEq/day) = 54.5 × protein (g/day)/potassium (mEq/day) − 10.2(1)

Protein and potassium intake was adjusted for total energy intake using the following equation:

Energy-adjusted nutrient intake = nutrient intake – b × (energy intake – mean of energy intake)(2)

where b is an estimated simple regression coefficient for the association between nutrient intake and total energy intake. Frassetto et al. obtained the above equation to estimate NEAP [18] from regression analysis using the steady-state rate of renal net acid excretion as a dependent variable, and the ratio of protein (acid precursor) to potassium (base precursor) content in the diet as an independent variable. 

### 2.5. Statistical Analysis

Continuous variables were shown as the medians and 25th and 75th percentiles, and categorical variables were expressed as counts and proportions. We examined the differences in participants’ characteristics according to NEAP scores using the Kruskal–Wallis test or chi-squared test.

Logistic-regression analysis was used to evaluate the associations of the quartiles of NEAP scores with the prevalence of MetS and its components after adjustment for potential confounding variables. The first quartile group was used as a reference to estimate odds ratios (ORs) and their profile likelihood 95% confidence intervals (CI). Model 1 was adjusted for age, sex, study center, total energy intake (quartile), physical activity (quartile), drinking habits (three categories), smoking habits (three categories), and school career (eight categories). Models 2–4 were additionally adjusted for Nutrient Pattern 1 scores (quartile), Nutrient Pattern 2 scores (quartile), and carbohydrate intake (quartile), respectively. Nutrient-pattern scores were calculated by factor analysis, as described in a previous report [25]. A factor-loading matrix for nutrient patterns is presented in Table S1. Trends across quartiles were examined by using ordinal categorical variables and a likelihood-ratio test. In addition, to examine the effect modification by sex, age, and BMI, stratified analysis was performed. Statistical significance of the interactions was examined by incorporating the product term of NEAP (continuous) and sex, age (≥55 and <55 years), or BMI (≥25 and <25 kg/m^2^) using a likelihood-ratio test. All data analyses were performed using FACTOR, LOGISTIC, and other procedures of SAS version 8.2.

## 3. Results

Table 1 shows the characteristics of the study subjects according to NEAP scores. Subjects with higher NEAP scores were more likely to be men, current or past smokers, current drinkers, and less physically active during leisure time. Participants with higher NEAP scores had higher BMI, systolic and diastolic blood pressure, serum triglycerides, and fasting blood glucose, and lower serum HDL cholesterol levels.

Table 1 also shows the median (with 25% and 75%) intake of nutrients and foods according to NEAP quartiles. NEAP scores were negatively correlated with carbohydrate, soluble and insoluble dietary fiber, n-6 polyunsaturated fatty acids, potassium, calcium, and sodium, and positively correlated with protein, fat, and cholesterol. Spearman’s rank correlation of NEAP scores with Nutrient Pattern 1 (fiber, iron, potassium, and vitamin pattern) and Nutrient Pattern 2 (fat and fat-soluble vitamins pattern) was −0.56 and 0.18, respectively. Regarding food intake, NEAP scores were positively correlated with fish, but negatively correlated with milk, yogurt, leafy green vegetables, green/yellow vegetables, cabbages, oranges, other fruits, and rice.

Table 2 shows the OR and 95% CI for the associations of NEAP scores with MetS and its components. After adjustment for sex, age, study site, smoking and drinking habits, physical-activity levels during leisure time, total energy intake, and school career (Model 1), higher NEAP scores were associated with significantly higher OR of MetS, obesity, high blood pressure, high serum triglycerides, and high blood glucose (*p* for trend < 0.001). After additional adjustment for Nutrient Pattern 1 scores (Model 2), associations were attenuated but remained statistically significant for MetS, obesity, high blood pressure, and high blood glucose (*p* for trend < 0.001). The OR of MetS in a subgroup with the highest NEAP scores (Q4), relative to that with the lowest scores (Q1), was 1.25 (95% CI 1.12–1.39). When Nutrient Pattern 2 scores (Model 3) or carbohydrate intake (Model 4) were adjusted instead of Nutrient Pattern 1 scores, results were similar to those in Model 1. In these analyses, Nutrient Pattern 1 and 2 scores were significantly associated with MetS, independent of NEAP scores (Table S2).

After stratifying by sex, age, or BMI, significant trends for the association between NEAP scores and MetS were observed for men and women, subjects aged ≥55 and <55 years, and obese subjects (≥25 kg/m^2^), with no significant effect modifications (Table 3). The trend for the association between NEAP scores and MetS did not greatly vary according to study site (Table S3).

## 4. Discussion

In the present study, there were significant positive trends between NEAP scores, and MetS and its components except for serum lipids. The associations remained significant after adjustment for carbohydrate intake and for two nutrient-pattern scores significantly associated with MetS in our previous study [25]. Results were similar after stratifying by sex, age, and BMI.

There have been very few studies on the relationship between dietary acid load and MetS, but Iwase et al. [19] reported that higher PRAL and NEAP scores were associated with a higher prevalence of MetS in 149 patients with Type 2 diabetes. In a cross-sectional study on 371 Iranian women, NEAP was significantly associated with large waist circumference and high serum triglyceride (TG) levels, but not with the prevalence of MetS (OR = 3.77, 95% CI 0.77–18.4) [20]. However, in these two studies, the number of subjects was small, and lifestyle factors such as smoking and drinking habits, physical activity, and dietary quality were not considered.

Positive relationships between dietary acid load and the risk of Type 2 diabetes were reported for women enrolled in the E3N-EPIC cohort [7], male participants in the JPHC study [8], and male and female health professionals in a meta-analysis of three cohort studies performed in the United States [9]. On the other hand, in a Swedish cohort study, neither PRAL nor NEAP was associated with the incidence rate of Type 2 diabetes mellitus (DM) in men [26]. Regarding other components of MetS, a significant association between higher serum anion gap and hypertension was reported in the National Health and Nutrition Examination Survey (NHENES) of the U.S. [27]. A prospective study also reported a positive trend between NEAP and the risk of hypertension in U.S. women [13], while a cross-sectional study showed a positive trend between NEAP and hypertension among Japanese workers with BMI < 23 kg/m^2^ or no shift work [14]. Recent meta-analysis of one prospective and eight cross-sectional studies showed a significant linear dose–response relationship between PRAL and hypertension [15]. However, the effect size was rather small, and the risk of hypertension associated with a 20 unit increase in PRAL was 1.03 (95% CI 1.00–1.06). In the National Health and Nutrition Survey of Japan, PRAL and NEAP (assessed on one-day weighted diet record) were positively correlated with BMI and systolic blood pressure, but not with serum HDL cholesterol or glycated hemoglobin concentrations [16]. In recently published meta-analysis, dietary acid load was positively associated with BMI/central obesity and serum triglycerides [28]. However, most studies included in this meta-analysis did not adjust for diet patterns, which are important confounders, as discussed later. Taken together, significant positive associations between dietary acid load and Type 2 DM, hypertension, obesity, and serum lipids have been reported by several researchers, but the results were not always consistent or conclusive.

Dietary acid load is positively correlated with a Western/high fat dietary pattern [7,9], and inversely correlated with a healthy/prudent dietary pattern [7]. In our study, NEAP scores were positively correlated with fat and fat-soluble vitamin pattern scores, while they were negatively correlated with fiber, iron, potassium, and vitamin pattern scores, both of which were significantly associated with the prevalence of MetS in our previous study [25]. Therefore, we adjusted for these nutrient pattern scores as potential confounders. After adjustment, the association between NEAP scores and MetS was attenuated, but still statistically significant. In these analyses, two nutrient patterns were significantly associated with MetS, independent of NEAP. The results suggested that the observed association between NEAP and MetS was not fully explained by healthy or high fat nutrition patterns, and that NEAP and these nutrition patterns reflected different aspects of dietary quality. 

Regarding underlying biological mechanisms, chronic low-grate metabolic acidosis is known to result in insulin resistance, an underlying condition of MetS. In the NHENES, serum bicarbonate concentration and serum anion gap were inversely and positively associated with fasting insulin levels and Homeostasis Model Assessment for Insulin Resistance (HOMA-IR), respectively [11]. The authors speculated that increased cortisol excretion mediated the relationship between metabolic acidosis and insulin resistance. In a cross-sectional study in a Japanese population, PRAL and NEAP were also positively associated with HOMA-IR [12]. Acidosis induces the transcription and expression of Transforming Growth Factor (TGF)-β, which inhibits insulin secretion and reduces the affinity of insulin to its receptor [29]. Acidosis also increases the urinary excretion of calcium and magnesium [30] that play important roles in insulin action [31,32,33]. These two minerals, together with potassium, reduce blood pressure by counterbalancing the effects of sodium [33]. Dietary calcium increases intracellular calcium concentration and reduces vascular resistance.

The strengths of the present study are the large number of study subjects, and adjustment for potential confounding factors, including nutrient patterns and school career. On the other hand, this study had several limitations. First, because of the cross-sectional study design, the temporal relationship between dietary acid load and MetS onset was not clear. Second, only NEAP was used as an index of dietary acid load. We could not calculate PRAL scores due to a lack of data on magnesium and phosphorus intake. However, in general, NEAP and PRAL scores have high positive correlation in Japanese populations (r ≥ 0.95) [8,14,16]. Third, because data on abdominal circumference were not available for every individual, BMI was used instead. When the BMI cut-off point of 25 kg/m^2^ was used, the sensitivity and specificity levels of our diagnosis of high waist circumference were 83% and 82% for men (no. = 633), and 45% and 98% for women (no. = 623), respectively, in the subsample of the J-MICC study in the Tokushima Prefecture [25]. Therefore, misclassification of abdominal obesity may have occurred, especially in women. However, misclassification may have been nondifferential with regard to NEAP scores, with the effect tending towards null results. Fourth, even though several potential confounders were adjusted, there may have been unknown confounding factors, as with all observational studies. Finally, since almost all the study participants were Japanese, the obtained results may not be directly applicable to other ethnic groups.

## 5. Concluisons

In conclusion, higher dietary acid load, as estimated by NEAP scores, was significantly associated with a higher prevalence of MetS and several of its components, independent of nutrient patterns significantly associated with MetS. Significant positive trends were observed irrespective of sex, age, and obesity. Further studies are required to clarify the temporal relationship between NEAP, and MetS and the underlying biological mechanisms.

## Figures and Tables

**Table 1:** Background characteristics of the study subjects according to their Net Endogenous Acid Production (NEAP) scores.

	Q1 (No. = 7036)	Q2 (No. = 7037)	Q3 (No. = 7037)	Q4 (No. = 7037)	*p*-Value ^b^
Age (years) ^a^	57 (48, 63)	56 (47, 63)	55 (46, 62)	54 (45, 62)	<0.001
Sex					
Men	2791 (39.7)	3486 (49.5)	3731 (53.0)	4034 (57.3)	<0.001
Women	4245 (60.3)	3551 (50.5)	3306 (47.0)	3003 (42.7)	
Smoking habit					
Current	1018 (14.5)	1252 (17.8)	1192 (16.9)	1227 (17.4)	<0.001
Past	1430 (20.3)	1637 (23.3)	1740 (24.7)	1929 (27.4)	
No	4588 (65.2)	4148 (59.0)	4105 (58.3)	3881 (55.2)	
Drinking habit					
Current	3654 (51.9)	4087 (58.1)	4247 (60.4)	4333 (61.6)	<0.001
Past	107 (1.5)	119 (1.7)	105 (1.5)	116 (1.7)	
No	3275 (46.6)	2831 (40.2)	2685 (38.2)	2588 (36.8)	
Exercise during leisure time (MET-hours/week)	7.65 (0.875, 20.85)	6.45 (0.425, 18)	5.1 (0.425, 17.85)	5.1 (0, 17.6)	<0.001
Body mass index (kg/m^2^)	22.4 (20.5, 24.6)	22.7 (20.7, 24.8)	22.9 (20.9, 25.1)	23.0 (21.0, 25.4)	<0.001
Systolic blood pressure (mmHg)	124 (111, 136)	124 (112, 136)	124 (113, 136)	126 (114, 138)	<0.001
Diastolic blood pressure (mmHg)	76 (68.5, 83)	76 (70, 84)	77 (70, 84)	78 (70, 85)	<0.001
Serum triglycerides (mg/dL)	89 (64, 127)	93 (67, 135)	95 (67, 137)	96 (68, 143)	<0.001
Serum HDL cholesterol (mg/dL)	64 (53, 76)	63 (52, 75)	62 (52, 74)	62 (51, 74)	<0.001
Fasting blood glucose (mg/dL)	93 (87, 100)	93 (88, 101)	94 (88, 102)	94 (88, 102)	<0.001
Energy intake(kcal/day)	1683 (1519, 1907)	1696 (1510, 1933)	1699 (1509, 1934)	1686 (1499, 1922)	0.03
Protein (g/day)	51 (45, 58)	52 (46, 58)	52 (46, 59)	54 (48, 61)	<0.001
Fat (g/day)	42 (35, 50)	42 (36, 49)	43 (36, 50)	44 (38, 52)	<0.001
Carbohydrate (g/day)	241 (212, 282)	240 (208, 285)	239 (207, 284)	234 (201, 276)	<0.001
Soluble dietary fiber (g/day)	2.2 (1.8, 2.6)	1.8 (1.5, 2.2)	1.7 (1.4, 2.1)	1.7 (1.4, 2.0)	<0.001
Insoluble dietary fiber (g/day)	8.7(7.2, 10.5)	7.4 (6.3, 8.7)	6.9 (6.0, 8.1)	6.6 (5.8, 7.6)	<0.001
Saturated fatty acids (g/day)	11(9, 13)	11 (9, 13)	11 (9, 13)	11 (9, 13)	0.32
Monounsaturated fatty acids (g/day)	16(13, 19)	15 (13, 18)	16 (13, 18)	16 (14, 19)	<0.001
*n*-3 polyunsaturated fatty acids (mg/day)	2164(1831, 2535)	2135 (1802, 2486)	2142 (1809, 2527)	2235 (1911, 2626)	<0.001
*n*-6 polyunsaturated fatty acids (mg/day)	11,058(9272, 13,241)	10,618 (9011, 12,658)	10,525 (8917, 12,596)	10,450 (8816, 12,571)	<0.001
Cholesterol (mg/day)	226(177, 276)	228 (184, 282)	235 (185, 288)	242 (195, 303)	<0.001
Potassium (mg/day)	2451(2184, 2763)	2116 (1915, 2337)	1928 (1736, 2131)	1718 (1539, 1916)	<0.001
Calcium (mg/day)	538 (440, 652)	503 (411, 601)	481 (394, 571)	444 (367, 534)	<0.001
Sodium (mg/day)	1719 (1473, 2065)	1680 (1437, 1971)	1665 (1419, 1947)	1658 (1389, 1949)	<0.001
Beef, pork (times/week)	1.5 (1.5, 3.5)	1.5 (1.5, 3.5)	1.5 (1.5, 3.5)	1.5 (1.5, 3.5)	<0.001
Fish (times/week)	1.5 (1.5, 3.5)	1.5 (1.5, 3.5)	3.5 (1.5, 3.5)	3.5 (1.5, 3.5)	<0.001
Milk (times/week)	3.5 (0.5, 7)	3.5 (0.5, 7)	1.5 (0.5, 7)	1.5 (0, 5.5)	<0.001
Yogurt (times/week)	1.5 (0.5, 5.5)	1.5 (0.5, 3.5)	1.5 (0, 3.5)	0.5 (0, 3.5)	<0.001
Egg (times/week)	3.5 (1.5, 3.5)	3.5 (1.5, 5.5)	3.5 (1.5, 5.5)	3.5 (1.5, 5.5)	<0.001
Green leafy vegetables (times/week)	3.5 (3.5, 7)	3.5 (1.5, 3.5)	1.5 (1.5, 3.5)	1.5 (0.5, 1.5)	<0.001
Green yellow vegetables (times/week)	3.5 (1.5, 5.5)	1.5 (1.5, 3.5)	1.5 (1.5, 3.5)	1.5 (0.5, 1.5)	<0.001
Cabbage (times/week)	3.5 (1.5, 5.5)	3.5 (1.5, 3.5)	1.5 (1.5, 3.5)	1.5 (1.5, 3.5)	<0.001
Radish (times/week)	1.5 (1.5, 3.5)	1.5 (1.5, 3.5)	1.5 (0.5, 1.5)	1.5 (0.5, 1.5)	<0.001
Orange (times/week)	1.5 (0.5, 5.5)	1.5 (0.5, 3.5)	1.5 (0.5, 3.5)	0.5 (0.5, 1.5)	<0.001
Other fruits (times/week)	3.5 (0.5, 5.5)	1.5 (0.5, 3.5)	1.5 (0.5, 3.5)	0.5 (0.5, 1.5)	<0.001
Rice (cups/week)	15.5 (12.5, 19.5)	14.5 (11.0, 19.5)	14.0 (11.0, 19.5)	14.0 (9.0, 16.5)	<0.001
Nutrient pattern scores					
Factor 1 (fiber, iron, potassium and vitamins pattern)	0.82 (0.18, 1.45)	0.10 (–0.48, 0.63)	–0.25 (–0.77, 0.29)	–0.64 (–1.15, –0.14)	<0.001
Factor 2 (fat and fat-soluble vitamins pattern)	–0.24 (–0.91, 0.44)	–0.18 (–0.79, 0.50)	–0.08 (–0.69, 0.62)	0.21 (–0.39, 0.91)	<0.001
NEAP (mEq/day)	35.2 (32.0, 37.4)	42.0 (40.6, 43.5)	47.5 (46.1, 49.1)	55.7 (52.9, 59.9)	-

Q, quartile; MET, metabolic equivalents; HDL, high-density lipoprotein. ^a^ Median (25%, 75%) or number of subjects (%). ^b^ Wilcoxon’s rank sum test or chi-square test.

**Table 2:** Associations of Net Endogenous Acid Production (NEAP) scores with metabolic syndrome and its components.

		Q1 (No. = 7036)	Q2 (No. = 7037)	Q3 (No. = 7037)	Q4 (No. = 7037)	
		OR	OR (95% CI)	OR (95% CI)	OR (95% CI)	*p* for Trend
Metabolic syndrome						
No. of cases (%)		977 (13.9)	1069 (15.2)	1171 (16.6)	1410 (20.0)	
Model 1 ^a^		1.00	1.03 (0.93–1.13)	1.12 (1.02–1.23)	1.39 (1.26–1.52)	<0.001
Model 2 ^b^		1.00	0.99 (0.90–1.09)	1.05 (0.95–1.16)	1.25 (1.12–1.39)	<0.001
Model 3 ^c^		1.00	1.02 (0.93–1.13)	1.11 (1.01–1.22)	1.35 (1.23–1.49)	<0.001
Model 4 ^d^		1.00	1.03 (0.94–1.13)	1.13 (1.02–1.24)	1.39 (1.27–1.53)	<0.001
Obesity						
No. of cases (%)		1482 (21.1)	1631 (23.2)	1786 (25.4)	1998 (28.4)	
Model 1		1.00	1.07 (0.98–1.16)	1.18 (1.08–1.27)	1.35 (1.24–1.46)	<0.001
Model 2		1.00	1.05 (0.97–1.14)	1.14 (1.04–1.24)	1.27 (1.16–1.39)	<0.001
Model 3		1.00	1.06 (0.98–1.15)	1.17 (1.07–1.26)	1.31 (1.21–1.42)	<0.001
Model 4		1.00	1.07 (0.98–1.16)	1.18 (1.09–1.28)	1.35 (1.25–1.47)	<0.001
High blood pressure						
No. of cases (%)		3124 (44.4)	3133 (44.5)	3325 (47.3)	3444 (48.9)	
Model 1		1.00	0.99 (0.92 – 1.06)	1.11 (1.03–1.19)	1.21 (1.12–1.30)	<0.001
Model 2		1.00	0.95 (0.88 – 1.03)	1.04 (0.96–1.13)	1.10 (1.01–1.19)	0.005
Model 3		1.00	0.98 (0.91 – 1.05)	1.09 (1.02–1.18)	1.18 (1.09–1.27)	<0.001
Model 4		1.00	0.98 (0.91 – 1.05)	1.10 (1.02–1.18)	1.18 (1.10–1.27)	<0.001
High serum triglycerides						
No. of cases (%)		1177 (16.7)	1366 (19.4)	1426 (20.3)	1619 (23.0)	
Model 1		1.00	1.07 (0.98–1.17)	1.08 (0.99–1.19)	1.21 (1.11–1.32)	<0.001
Model 2		1.00	1.01 (0.92–1.11)	1.00 (0.91–1.10)	1.08 (0.98–1.20)	0.13
Model 3		1.00	1.06 (0.97–1.16)	1.08 (0.99–1.18)	1.21 (1.10–1.32)	<0.001
Model 4		1.00	1.07 (0.98–1.17)	1.09 (1.00–1.19)	1.22 (1.12–1.33)	<0.001
Low HDL cholesterol						
No. of cases (%)		637 (9.1)	600 (8.5)	569 (8.1)	595 (8.5)	
Model 1		1.00	0.99 (0.88–1.12)	0.97 (0.86–1.09)	1.05 (0.93–1.19)	0.54
Model 2		1.00	0.95 (0.84–1.07)	0.90 (0.79–1.03)	0.96 (0.84–1.11)	0.49
Model 3		1.00	0.99 (0.88–1.12)	0.97 (0.85–1.09)	1.05 (0.93–1.19)	0.53
	Model 4	1.00	1.02 (0.91–1.15)	1.01 (0.89–1.14)	1.14 (1.01–1.29)	0.06
High blood glucose						
No. of cases (%)		1893 (26.9)	2024 (28.8)	2161 (30.7)	2304 (32.7)	
Model 1		1.00	1.04 (0.96–1.12)	1.14 (1.05–1.23)	1.26 (1.17–1.36)	<0.001
Model 2		1.00	1.01 (0.93–1.10)	1.09 (1.00–1.18)	1.17 (1.07–1.28)	<0.001
Model 3		1.00	1.04 (0.96–1.12)	1.13 (1.05–1.23)	1.25 (1.16–1.36)	<0.001
Model 4		1.00	1.04 (0.96–1.12)	1.13 (1.05–1.22)	1.25 (1.15–1.35)	<0.001

Q, quartile; OR, odds ratio; CI, confidence interval; HDL, high-density lipoprotein. ^a^ Adjusted for sex, age, study site, smoking and drinking habits, physical activity level, total energy intake, and school career. ^b^ Model 1 plus nutrient pattern 1 (fiber, iron, potassium and vitamins pattern) scores. ^c^ Model 1 plus nutrient pattern 2 (fat and fat-soluble vitamins pattern) scores. ^d^ Model 1 plus carbohydrate intake.

**Table 3:** Associations of Net Endogenous Acid Production (NEAP) scores with metabolic syndrome according to sex, age and body mass index (BMI).

	OR	OR (95% CI)	OR (95% CI)	OR (95% CI)	*p* for Trend	*p* Interaction
Men	Q1 (No. = 3510)	Q2 (No. = 3511)	Q3 (No. = 3510)	Q4 (No. = 3511)		
No. of cases (%)	721 (20.5)	723 (20.6)	786 (22.4)	925 (26.3)		
Model 1 ^a^	1.00	1.00 (0.89–1.12)	1.11 (0.99–1.24)	1.38 (1.23–1.55)	<0.001	0.61 ^d^
Model 2 ^b^	1.00	0.96 (0.85–1.08)	1.03 (0.91–1.17)	1.23 (1.08–1.40)	<0.001	0.75
Women^c^	Q1 (No. = 3526)	Q2 (No. = 3526)	Q3 (No. = 3526)	Q4 (No. = 3527)		
No. of cases (%)	349 (9.9)	351 (10.0)	344 (9.8)	428 (12.1)		
Model 1	1.00	1.04 (0.89–1.22)	1.02 (0.87–1.20)	1.41 (1.20–1.64)	<0.001	
Model 2	1.00	0.98 (0.83–1.16)	0.93 (0.78–1.11)	1.21 (1.01–1.46)	0.05	
Age <55 years	Q1 (No. = 3342)	Q2 (No. = 3342)	Q3 (No. = 3342)	Q4 (No. = 3342)		
No. of cases (%)	363 (10.9)	415 (12.4)	453 (13.6)	514 (15.4)		
Model 1	1.00	1.03 (0.88–1.20)	1.12 (0.96–1.31)	1.30 (1.12–1.52)	<0.001	0.44 ^e^
Model 2	1.00	1.00 (0.85–1.17)	1.06 (0.90–1.25)	1.19 (1.01–1.41)	0.02	0.33
Age ≥55 years	Q1 (No. = 3694)	Q2 (No. = 3695)	Q3 (No. = 3695)	Q4 (No. = 3695)		
No. of cases (%)	582 (15.8)	675 (18.3)	708 (19.2)	917 (24.8)		
Model 1	1.00	1.08 (0.96–1.23)	1.11 (0.98–1.26)	1.46 (1.29–1.65)	<0.001	
Model 2	1.00	1.03 (0.91–1.18)	1.02 (0.89–1.17)	1.30 (1.13–1.49)	<0.001	
BMI < 25 kg/m^2^	Q1 (No. = 5312)	Q2 (No. = 5313)	Q3 (No. = 5312)	Q4 (No. = 5313)		
No. of cases (%)	242 (4.6)	266 (5.0)	245 (4.6)	348 (6.5)		
Model 1	1.00	1.04 (0.87–1.25)	0.95 (0.79–1.14)	1.37 (1.15–1.63)	0.001	0.34 ^f^
Model 2	1.00	1.00 (0.83–1.21)	0.88 (0.72–1.07)	1.22 (1.00–1.50)	0.08	0.34
BMI ≥ 25 kg/m^2 c^	Q1 (No. = 1724)	Q2 (No. = 1724)	Q3 (No. = 1724)	Q4 (No. = 1725)		
No. of cases (%)	843 (48.9)	835 (48.4)	917 (53.2)	931 (54.0)		
Model 1	1.00	0.95 (0.83–1.09)	1.13 (0.98–1.29)	1.19 (1.03–1.37)	0.003	
Model 2	1.00	0.93 (0.81–1.08)	1.09 (0.94–1.27)	1.14 (0.97–1.33)	0.03	

OR, odds ratio; CI, confidence interval; Q, quartile. ^a^ Adjusted for age, study site, smoking and drinking habits, physical activity level, total energy intake, and school career. ^b^ Model 1 plus nutrient pattern 1 (fiber, iron, potassium and vitamins pattern) scores. ^c^ Physical activity was classified into 3 groups (Q1+Q2, Q3, and Q4). ^d^ *p* for interaction between NEAP scores (continuous) and sex. ^e^ *p* for interaction between NEAP scores (continuous) and age (dichotomous). ^f^ *p* for interaction between NEAP scores (continuous) and BMI (dichotomous).

## Supplementary Materials

The following are available online at https://www.mdpi.com/2072-6643/12/6/1605/s1, Table S1: Factor loading matrix for nutrient patterns, Table S2: Associations of nutrient patterns 1 and 2 scores with metabolic syndrome, adjusted for Net Endogenous Acid Production (NEAP) scores, Table S3: Associations of Net Endogenous Acid Production (NEAP) scores with metabolic syndrome according to study site.
