# Supplementary material for: Association of Dietary Acid Load with the Prevalence of Metabolic Syndrome among Participants in Baseline Survey of the Japan Multi-Institutional Collaborative Cohort Study
Source: Nutrients. 2020 May 29;12(6):1605. doi: 10.3390/nu12061605 (PMC7352218; doi:10.3390/nu12061605)
Supplement: Supplementary file 1 [file nutrients-12-01605-s001.pdf]

Table S1. Factor loading matrix for nutrient patterns.

	Factor 1 [a]	Factor 2 [b]	Factor 3
Protein[c]	0.36	0.43	0.64
Carbohydrate	0.09	-0.28	-0.59
Soluble dietary fiber	0.82	0.19	0.19
Insoluble dietary fiber	0.84	0.22	0.17
Saturated fatty acids	0.13	0.17	0.86
Monounsaturated fatty acids	0.12	0.92	0.17
n-3 polyunsaturated fatty acids	0.24	0.86	0.16
n-6 polyunsaturated fatty acids	0.28	0.80	0.07
Cholesterol	0.15	0.40	0.39
Potassium	0.76	0.15	0.31
Calcium	0.45	-0.05	0.75
Iron	0.81	0.19	0.17
Retinol	0.65	0.19	0.11
Carotene	0.82	0.23	0.06
Vitamin D	0.40	0.35	0.27
Vitamin E	0.49	0.78	0.09
Vitamin B_1	0.10	0.55	0.43
Vitamin B_2	0.52	0.04	0.71
Vitamin C	0.80	0.13	0.08
Folate	0.88	0.17	0.06

Sodium	0.47	0.14	0.17
Cumulative variance explained	0.45	0.57	0.66

[a] Named the fiber, iron, potassium and vitamins pattern.

[b] Named the fat and fat-soluble vitamins pattern.

[c] Nutrient intake was adjusted for energy intake using the residual method.

Table S2. Associations of nutrient pattern 1 and 2 scores with metabolic syndrome, adjusted for Net Endogenous Acid Production (NEAP) scores.

	OR [a]	OR (95% CI)	OR (95% CI)	OR (95% CI)	*p* for trend
Nutrient pattern 1 scores	Q1 (No. = 7036)	Q2 (No. = 7037)	Q3 (No. = 7037)	Q4 (No. = 7037)	
No. of cases (%)	1441 (20.5)	1171 (16.6)	1060 (15.1)	955 (13.6)	
(Fiber, iron, potassium and vitamins pattern)	1.00	0.83 (0.76 – 0.91)	0.80 (0.73 – 0.88)	0.77 (0.68 – 0.86)	< 0.001
Nutrient pattern 2 scores	Q1 (No. = 7036)	Q2 (No. = 7037)	Q3 (No. = 7037)	Q4 (No. = 7037)	
No. of cases (%)	1194 (17.0)	1175 (16.7)	1064 (15.1)	1194 (17.0)	
(Fat and fat-soluble vitamins pattern)	1.00	1.09 (1.00 – 1.20)	1.02 (0.93 – 1.12)	1.19 (1.08 – 1.31)	0.003

OR, odds ratio; CI, confidence interval; Q, quartile.

[a] Adjusted for sex, age, study site, smoking and drinking habits, physical activity level, total energy intake, school career, and NEAP scores.

Table S3. Associations of Net Endogenous Acid Production (NEAP) scores with metabolic syndrome according to study site.

	OR [a]	OR (95% CI)	OR (95% CI)	OR (95% CI)	*p* for trend [b]
Study site 1	Q1 (No. = 1458)	Q2 (No. = 1459)	Q3 (No. = 1459)	Q4 (No. = 1459)	
No. of cases (%)	193 (13.2)	209 (14.3)	228 (15.6)	262 (18.0)	
Model 2	1.00	1.00 (0.80 – 1.25)	1.04 (0.83 – 1.32)	1.14 (0.90 – 1.46)	0.23
Study site 2	Q1 (No. = 1126)	Q2 (No. = 1126)	Q3 (No. = 1126)	Q4 (No. = 1127)	
No. of cases (%)	134 (11.9)	167 (14.8)	171 (15.2)	210 (18.6)	
Model 2	1.00	1.11 (0.86 – 1.45)	1.10 (0.83 – 1.45)	1.35 (1.02 – 1.79)	0.04
Study site 3 [c]	Q1 (No. = 464)	Q2 (No. = 464)	Q3 (No. = 464)	Q4 (No. = 464)	
No. of cases (%)	55 (11.9)	64 (13.8)	50 (10.8)	76 (16.4)	
Model 2	1.00	1.23 (0.80 – 1.90)	0.86 (0.54 – 1.37)	1.45 (0.92 – 2.31)	0.26
Study site 4	Q1 (No. = 1292)	Q2 (No. = 1292)	Q3 (No. = 1292)	Q4 (No. = 1293)	
No. of cases (%)	160 (12.4)	169 (13.1)	183 (14.2)	261 (20.2)	
Model 2	1.00	0.96 (0.75 – 1.23)	0.94 (0.73 – 1.21)	1.31 (1.02 – 1.70)	0.03
Study site 5 [d]	Q1 (No. = 1413)	Q2 (No. = 1414)	Q3 (No. = 1413)	Q4 (No. = 1414)	
No. of cases (%)	259 (18.3)	317 (22.4)	317 (22.4)	366 (25.9)	
Model 2	1.00	1.14 (0.93 – 1.38)	1.08 (0.88 – 1.33)	1.20 (0.97 – 1.48)	0.17
Study site 6	Q1 (No. = 278)	Q2 (No. = 278)	Q3 (No. = 278)	Q4 (No. = 278)	
No. of cases (%)	34 (12.2)	45 (16.2)	45 (16.2)	57 (20.5)	
Model 2	1.00	1.28 (0.76 – 2.19)	1.24 (0.72 – 2.16)	1.68 (0.97 – 2.93)	0.08

Study site 7	Q1 (No. = 1004)	Q2 (No. = 1004)	Q3 (No. = 1004)	Q4 (No. = 1004)	
No. of cases (%)	123 (12.3)	107 (10.7)	167 (16.6)	198 (19.7)	
Model 2	1.00	0.73 (0.54 – 0.97)	1.16 (0.88 – 1.54)	1.35 (1.01 –1.82)	0.002

OR, odds ratio; CI, confidence interval; Q, quartile.

[a] Adjusted for sex, age, smoking and drinking habits, physical activity level, total energy intake, school career, and nutrient pattern 1 scores.

[b] By Wald test.

[c] Physical activity was used as a continuous variable.

[d] Physical activity was classified into 3 groups (Q1+Q2, Q3, Q4).
